# The Effect of Local Papaverine Use in an Experimental High-Risk Colonic Anastomosis Model: Reduced Inflammatory Findings and Less Necrosis

**DOI:** 10.3390/jcm13185638

**Published:** 2024-09-23

**Authors:** Dursun Burak Ozdemir, Ahmet Karayigit, Emel Tekin, Evin Kocaturk, Cengiz Bal, Ilter Ozer

**Affiliations:** 1 Department of Surgical Oncology, SBU Samsun Training and Research Hospital, 55090 Samsun, Turkey; 2 Department of Surgical Oncology, Dr. Abdurrahman Yurtaslan Ankara Oncology Training and Research Hospital, 06200 Ankara, Turkey; drkarayigitahmet@gmail.com; 3 Department of Pathology, Faculty of Medicine, Eskişehir Osmangazi University, 26480 Eskisehir, Turkey; emelyaldir@gmail.com; 4 Department of Medical Biochemistry, Faculty of Medicine, Eskişehir Osmangazi University, 26480 Eskisehir, Turkey; drekocaturk@gmail.com; 5 Department of Biostatistics, Faculty of Medicine, Eskişehir Osmangazi University, 26480 Eskisehir, Turkey; cengiz@ogu.edu.tr; 6 Department of Gastroenterology Surgery, Private Office, 06560 Ankara, Turkey; ilterozer@yahoo.com

**Keywords:** colonic anastomosis, papaverine, anastomotic leak, wound healing, experimental study

## Abstract

**Objectives:** To assess the impact of topical papaverine administration in complete and incomplete colonic anastomosis, by examining bursting pressure, hydroxyproline concentration, collagen content, inflammation levels, inflammatory cell infiltration, neoangiogenesis, and necrosis grades. **Methods:** We performed an experimental study on rats, in which they were divided into the following 4 groups of 16 subjects each. Group 1 [complete anastomosis (CA) without papaverine (CA -P) group], Group 2 [CA with papaverine (CA +P) group], Group 3 [incomplete anastomosis (ICA) without papaverine (ICA -P) group], and Group 4 [ICA with papaverine (ICA +P) group]. **Results:** The lymphocyte infiltration score of the ICA +P3 (day 3) group was significantly higher compared to the ICA -P3 group (*p* = 0.018). The median Ehrlich–Hunt score (*p* = 0.012), inflammation score (*p* = 0.026), and neutrophil infiltration score (*p* = 0.041) of the CA +P7 (day 7) group were significantly lower than the corresponding data of the CA -P7 group. Additionally, the necrosis score of the ICA +P7 group was significantly lower than that of the ICA -P7 group (*p* = 0.014). **Conclusions:** Data from the current study reveal that, although topical papaverine seems to suppress inflammation in anastomosis tissue and reduce necrosis at 7 days, definite conclusions regarding its impact on anastomotic leak cannot be drawn without further studies investigating anastomotic wound healing and anastomotic leak, preferably with both shorter- and longer-term evaluations.

## 1. Introduction

Anastomotic leak (AL) has diverse clinical and radiological characteristics, ranging from radiological signs to severe complications like peritonitis and sepsis. It affects surgical outcomes, prolongs hospital stay, increases costs, and worsens prognosis [1]. AL demonstrates an incidence of 2% to 19% after colorectal surgery [2,3,4] and results in mortality in 10% to 32% of cases [5,6,7].

Despite the utilization of various surgical techniques and preventive methods in recent years, AL still continues to be an important problem [8,9]. The implemented surgical technique appears to have minimal impact on AL incidence [8]. New methods have been employed to help complete the anastomosis, including direct fibrin glue application [8,10], use of fibrin glue-coated collagen patches [11], and hydrogel adhesive [12]. However, conflicting results have been reported regarding their success, and many of these techniques do not improve wound healing or tissue vascularity [8,10,13].

For successful anastomosis, sufficient wound healing is paramount. Even if the surgical procedure is performed perfectly, AL is highly likely if wound healing is impaired [8]. Optimal tissue perfusion and sufficient oxygen supply are necessary to ensure good wound healing and anastomosis safety [14]. Papaverine, an opium alkaloid, is a non-selective phosphodiesterase inhibitor that causes direct vasodilation and prevents reflex vasospasm, thereby improving circulation. It acts on both arteries and veins and has been shown to contribute to the formation of collateral circulation in venous insufficiency [11,15]. As such, papaverine exerts relaxing and antispasmodic effects on smooth muscles in vessels and the gastrointestinal, urinary, and biliary systems [16]. Numerous studies have investigated the effects of this nonspecific vasodilator in vascular diseases, such as nonocclusive mesenteric ischemia, cerebral vasospasm, angina pectoris, Reynaud’s phenomenon, Buerger’s disease, and perioperative arterial spasm, and also it has been explored for its utility in preventing ischemia in skin and muscle grafts [15,16,17,18,19,20]. However, relatively few studies have investigated the effect of papaverine in colon anastomosis [11,21]. In these studies, treatment with repeated doses of intraperitoneal papaverine was investigated; however, to our knowledge, the effect of a single dose of papaverine directly applied on the anastomosis has not been investigated.

We postulated that the topical application of papaverine to areas of complete and/or incomplete colon anastomosis may contribute to anastomosis healing. Therefore, in this experimental high-risk colonic anastomosis model in rats, we aimed to compare various critical parameters between rats undergoing complete or incomplete colon anastomosis with and without topical papaverine administration, thereby attempting to clarify the effects of papaverine on anastomotic wound healing. The parameters examined were bursting pressure, hydroxyproline concentration, collagen content, inflammation degree, inflammatory cell infiltration, neoangiogenesis and necrosis grades.

## 2. Materials and Methods

### 2.1. Ethical Statement

The current study was approved by the Local Animal Experiments Ethics Committee of Eskişehir Osmangazi University (Decision date: 30 June 2021, decision no: 846).

### 2.2. Study Setting, Animals, and Grouping

This experimental study was carried out by the Gastroenterology Surgery Department of Osmangazi University Faculty of Medicine, from December 2022 to May 2023.

A total of 72 Wistar albino rats, 300–350 g in weight, 3–4 months old, randomly selected from a group of healthy male subjects, were used in the study. All rats were housed in conditions with unrestricted access to water and food, a room temperature of 22–24 °C, and 12 h light and dark cycles.

The rats were divided into the following 4 groups of 16 subjects each. Group 1 [complete anastomosis (CA) without papaverine (CA -P) group]—these rats underwent complete colon anastomosis without papaverine administration. Group 2 [CA with papaverine (CA +P) group], which included rats that underwent complete colon anastomosis with papaverine administration. Group 3 [incomplete anastomosis (ICA) without papaverine (ICA -P) group] included rats that underwent defective anastomosis without papaverine administration. Group 4 [ICA with papaverine (ICA +P) group] included rats that underwent defective anastomosis with papaverine administration. Each group was divided into two subgroups based on the timing of sacrifice (day 3 and day 7). Thus, a total of 8 groups, each consisting of 8 rats, were created. The abbreviated names of the groups were as follows: CA -P3, CA -P7, CA +P3, CA +P7, ICA -P3, ICA -P7, ICA +P3, and ICA +P7.

### 2.3. Anesthesia, Surgery, and Euthanasia

**Figure 1:**
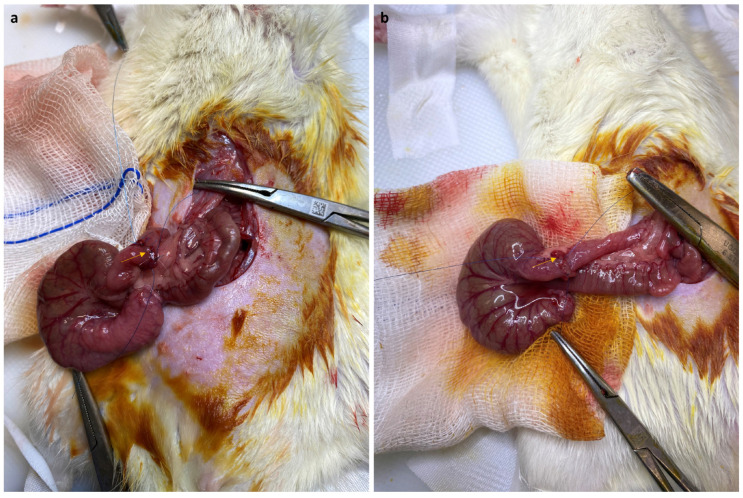
Intraoperative images of complete anastomosis (**a**) and incomplete anastomosis (**b**).

The same pre-anesthesia, anesthesia, pre-anastomosis, and early post-anastomosis procedures were utilized in all groups. Anesthesia was administered using 60 mg/kg intramuscular ketamine and 10 mg/kg xylazine. The animals were allowed to breathe spontaneously during surgery. A heating lamp was used to keep body temperatures around 37 °C. At the end of the operation, 10 mL of Ringer’s lactate solution was administered subcutaneously to prevent dehydration. Antibiotic prophylaxis was applied by intramuscular administration of 30 mg/kg ceftriaxone 30 min before the incision. After shaving the abdomen, the surgical area was cleaned twice with copious 10% povidone iodide solution, and the operation was carried out under sterile conditions. The peritoneal cavity was opened with a midline incision made under the xiphoid, and the cecum was exposed. A complete transection of the colon was performed 2 cm from the floor of the cecum without resection. In Group 1 and Group 2, both lumens were sutured end-to-end with 8 interrupted sutures (6/0 polypropylene; Prolene, Ethicon, Inc., Somerville, NJ, USA) (Figure 1a). After this complete anastomosis, papaverine (Papaverine HCl, 60 mg/kg, Galen Ilac AS; Istanbul, Turkey) was administered at a dose of 60 mg/kg in the form of a spray, covering approximately 1 cm distal and proximal to the anastomosis, using a 26-gauge needle tip in Group 2, while no papaverine was applied to Group 1. In Group 3 and Group 4, both lumens were sutured end to end with 6/0 polypropylene suture material with 4 interrupted sutures (Figure 1b). After this incomplete anastomosis, papaverine was applied by spraying in Group 4 similarly, but it was not applied in Group 3. In all groups, the abdominal incisions were closed over two layers with 3/0 polyglactin (Vicryl, Ethicon, Inc., Somerville, NJ, USA). In the early postoperative period, 1–2 mg/kg meloxicam was administered subcutaneously as an analgesic. No water was allowed for the first 4 h after the operation, and then free access was provided. Daily observations of the health status of the rats were performed. After resuming oral feeding, all animals were given a liquid diet (10% dextrose) for 3 days. The subgroups of rats that were euthanized on day 3 were selected randomly. The remaining rats received standard rodent chow for the next 4 days, and these rats were euthanized on the 7th day. Euthanasia was performed after complete anesthesia via intracardiac puncture and complete blood withdrawal.

### 2.4. Data Collection Tools

For all rats (both euthanized on day 3 and on day 7), the following parameters were analyzed: weight, anastomotic bursting pressure, histopathological Erlich–Hunt score, inflammation, fibroblast activity, neoangiogenesis, necrosis, collagen deposition, infiltration of neutrophils and lymphocytes, and hydroxyproline concentrations of the intestinal segment containing the anastomotic tissue.

#### 2.4.1. Anastomotic Bursting Pressure Measurement

Anastomotic burst pressures were measured immediately after the rats were sacrificed. Re-laparotomy was performed, and a 4 cm segment was dissected, including the anastomotic site, the cecum, and the ascending colon. The excised segment was freed from any stool and particulate matter, and the colonic ends were sutured with 3-0 silk to close the colonic lumen. An 18-gauge arterial catheter was inserted into the ascending colon from the distal end, and the insertion site was fastened with two 3/0 silk sutures. The other end of the catheter was connected to an infusion pump (Infusomat^®^, Braun, Melsungen, Germany) that administered isotonic saline at a rate of 2 mL/min. Intraluminal pressure was measured and recorded using an amplified pressure transducer that recorded values in mmHg (Viridea M3 Monitor HP M3046A, Hewlett Packard, Boeblingen, Germany). Anastomotic burst pressure was defined as the maximum intraluminal pressure measured before leakage occurred [11,22].

#### 2.4.2. Histopathological Assessment

**Figure 2:**
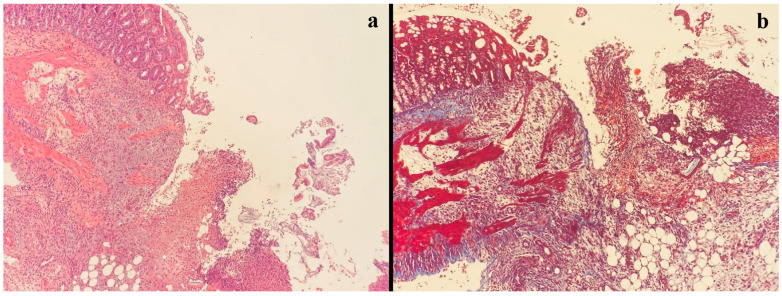
In the case with fibroblast activity grade 0, collagen was not observed with Masson’s trichrome histochemical stain, showing poor angiogenesis, intense edema, and neutrophilic inflammation (**a**) H&E staining, (**b**) Masson’s trichrome staining.

**Figure 3:**
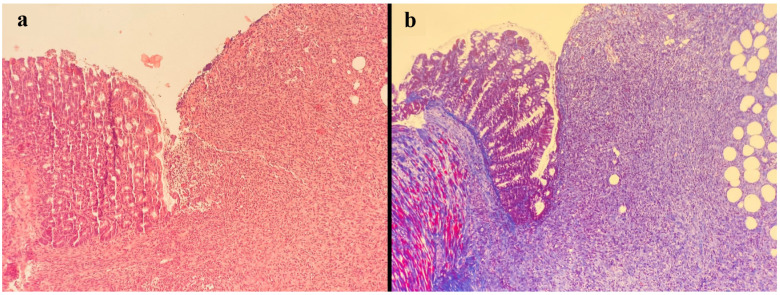
In the case of fibroblast activity grade 4, intense collagen fibril formation was observed with Masson’s trichrome histochemical stain, indicating rich angiogenesis, minimal edema, and intense lymphocytic inflammation (**a**) H&E staining, (**b**) Masson’s trichrome staining.

All histopathological examinations were performed by the same pathologist with standard methods in the Pathology Department of Eskişehir Osmangazi University. Briefly, after resection, the anastomosis site was stored in 10% buffered formaldehyde solution for 24 h, at which point it was embedded in paraffin. The 4 µm sections were stained with hematoxylin and eosin and Masson’s trichrome (Figure 2 and Figure 3). Anastomoses were graded histologically using the modified Ehrlich–Hunt scale [23]. Inflammation, fibroblast activity, neoangiogenesis, necrosis, collagen deposition, and infiltration of neutrophils and lymphocytes were graded from 0 to 4 (0: none, 1: occasional, 2: lightly scattered, 3: abundant, 4: merging cells or fibers) [22].

#### 2.4.3. Hydroxyproline Concentration

Hydroxyproline concentrations were measured by the Department of Medical Biochemistry. A 1 cm colon segment containing the anastomosis was resected and rinsed in PBS. The samples were weighed, homogenized, and sonicated. Then, the homogenates were centrifuged at 10,000× *g* for 5 min, after which supernatants were collected and hydroxyproline levels were measured in these supernatants with enzyme-linked immunosorbent assay kits (catalog no: CEA621ge, USCN lifescience, Wuhan, China), according to the manufacturer’s instructions, and recorded as nanograms per mL (ng/mL) [11,22].

### 2.5. Statistical Analysis

The classical significance threshold was used for all hypothesis testing (two-tailed *p* values of less than 0.05). All analyses were performed on IBM SPSS for Windows, Version 21.0 (IBM Corp., Armonk, NY, USA). Histograms and Q-Q plots were used to determine the normality of distribution, which showed that the best descriptive method for continuous data was to calculate median (minimum–maximum) values. Due to the same reason, continuous data were compared with the Mann–Whitney U test. Frequency and percentage values were used to summarize categorical (ordinal) variables. Categorical data were compared with the Fisher’s exact test or the Fisher–Freeman–Halton test.

## 3. Results

**Figure 4:**
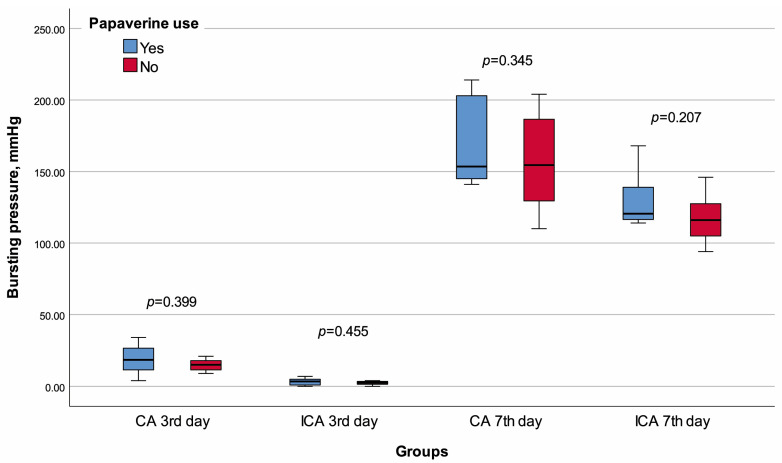
Box plot of the bursting pressure with regard to groups and papaverine use.

**Figure 5:**
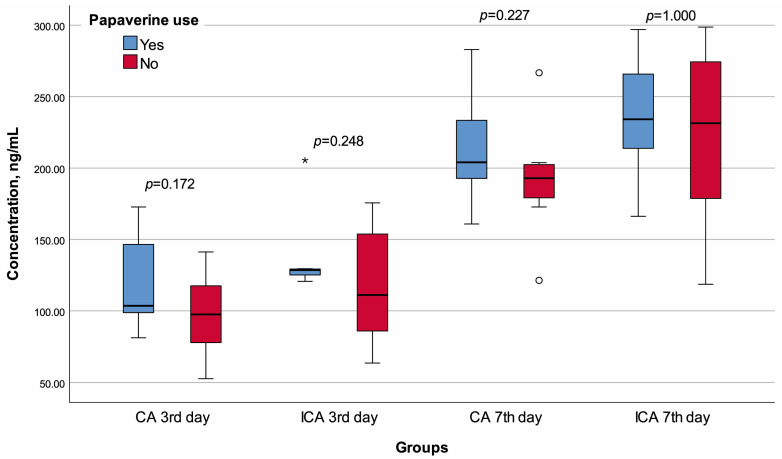
Box plot of the hydroxyproline concentration with regard to groups and papaverine use. o Mild outlier, * Extreme outlier.

**Figure 6:**
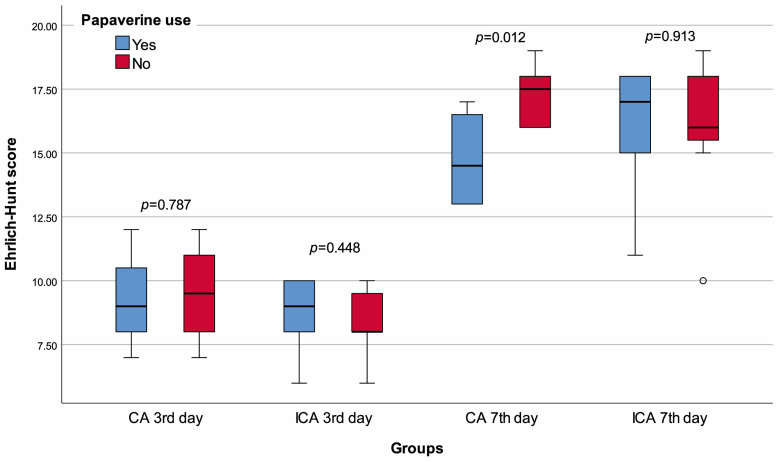
Box plot of the Ehrlich–Hunt score with regard to groups and papaverine use. o Mild outlier.

None of the rats died before planned sacrifice, and no visible leakage was observed when their abdomens were opened. Rats in the CA -P3 group were significantly heavier than those in the CA +P3 group (*p* = 0.002). There was no significant difference between CA +P3 and CA -P3 in terms of any of the other variables (Table 1, Figure 4, Figure 5 and Figure 6).

The lymphocyte infiltration score of the ICA +P3 group was significantly higher than that of the ICA -P3 group (*p* = 0.018). There was no significant difference between the ICA +P3 and ICA -P3 groups in terms of any other variables (Table 2, Figure 3, Figure 4 and Figure 5).

The median Ehrlich–Hunt (*p* = 0.012), inflammation (*p* = 0.026), and neutrophil infiltration (*p* = 0.041) scores of the CA +P7 group were significantly lower than that of CA -P7 group. The CA +P7 and CA -P7 groups had similar values for other variables (Table 3, Figure 3, Figure 4 and Figure 5).

The necrosis score of the ICA +P7 group was significantly lower than that of the ICA -P7 (*p* = 0.014). Again, all other comparisons demonstrated similar results in the ICA +P7 and ICA -P7 groups (Table 4, Figure 3, Figure 4 and Figure 5).

## 4. Discussion

In the case of leakage of luminal contents to the abdominal region due to colonic AL, patients may experience fever, abscess, septicemia, metabolic disorders, or multi-organ failure, which may increase the risk of reoperation, local recurrence, morbidity, and mortality, as well as leading to a decrease in quality of life [8,24]. Therefore, it is crucial to develop new interventions that can improve the healing of colon anastomosis. In this experimental study, we investigated the benefit of topical papaverine administration in colonic anastomosis healing. The results of this study showed that the lymphocyte cell infiltration score of the ICA +P3 group was significantly higher than that of ICA -P3 group. Ehrlich–Hunt score, inflammation score, and neutrophil infiltration score of the CA +P7 group were significantly lower compared to CA -P7. The necrosis score of ICA +P7 was significantly lower than that of ICA -P7.

Optimal tissue perfusion is necessary for faster intestinal anastomosis healing [14]. In a systematic review published in 2016, hyperbaric oxygen was identified as being the only useful intervention that improved burst pressure values in rat models of anastomotic ischemia [25]. Also, studies have reported lower rates of wound infection with an inspired oxygen fraction of 80% versus 30% [14,26]. Papaverine induces vasodilation in the circulation and prevents reflex vasospasm [11]. In several vascular diseases, papaverine’s benefit has been investigated [15,16,17,18,19,20]. For instance, the effects of papaverine on arterial spasm in patients who had undergone coronary artery bypass surgery have been investigated, showing that perivascular administration of papaverine increased blood flow [18]. Our primary aim was to investigate the effect of papaverine on intestinal anastomosis healing by examining crucial parameters. Papaverine did not improve bursting pressure, hydroxyproline concentration, neoangiogenesis, and collagen deposition in neither the ICA nor CA groups at 3 or 7 days. However, papaverine significantly improved lymphocyte score after 3 days in the ICA group; the degree of necrosis after 7 days in the ICA group; and the Ehrlich–Hunt score, inflammation score, and neutrophil score after 7 days in the CA group. In a similar experimental study in which papaverine was tried in colon anastomosis healing, one group received intraperitoneal papaverine before abdominal closure, and another group was administered 60 mg/kg papaverine intraperitoneally daily for an additional 10 days. Compared to controls, there were significant differences in papaverine recipients in terms of anastomotic burst pressure, hydroxyproline measurements, and macroscopic adhesion degree, all of which favored papaverine use [11]. In the mentioned study, all groups underwent end-to-end colonic anastomosis after colon transection. In our study, some groups underwent complete colonic anastomosis, while others underwent incomplete anastomosis. This allowed for a more unbiased evaluation of the direct effects of papaverine on anastomotic healing. In our study, measurements were made on both the third and seventh days, enabling an assessment of the temporal effects of the variables. Additionally, our study assessed not only macroscopic outcomes but also histological and biochemical parameters, providing a deeper insight into anastomotic healing. Furthermore, the authors of the prior study administered papaverine intraperitoneally [11], whereas we chose to apply the treatment directly to the anastomosis site. The differences in papaverine-related outcomes between the studies may suggest that the method of administration plays a significant role. In another experimental study, which investigated the effect of intraperitoneal papaverine on the levels of vascular endothelial growth factor, it was reported that papaverine had no significant effect on factor levels measured 10 days after anastomosis [21].

Anastomotic leakage is an outcome caused by surgical intervention; however, it is also associated with host genetics, gut microbiome, inflammation, and immune response [27]. Failure in anastomotic healing and leakage of intestinal contents can have devastating consequences for patients undergoing intra-abdominal surgery [28]. The rate of collagen synthesis and remodeling is faster in ileal anastomosis than in colonic anastomosis [29]. In addition, small bowel anastomoses are reported to reach near-normal strength at four weeks postoperatively, while colonic anastomoses only regain 75% of normal tissue strength at four months. These differences in healing may be due to differences in collagenase activity immediately after surgery. The risk of AL appears to be higher in colonic anastomoses compared to those in the small intestine [28].

Considering the high risks associated with AL, many studies have been conducted on interventions to improve anastomotic healing in animal models. Most of these studies focus on colonic anastomosis. An experimental study by Cakir et al. reported that oral sildenafil treatment increased bursting pressures, collagen maturity, collagen content, and epithelization score and decreased malondialdehyde levels, neutrophil score, and inflammation score [22]. Another experimental study investigated the effect of fibrin-glue-coated collagen patches on the healing process of colonic anastomoses in rats with unfavorable healing processes (technical deficiencies and peritonitis). As a result, the beneficial effect of additional gluing with a fixed combination of collagen matrix-bound coagulation factors I and IIa was revealed. Bonding of incomplete anastomoses resulted in significantly lower leak rates and death, higher burst pressure values, and histopathological scores. Collagen 1 and 3 expressions and hydroxyproline concentrations were greatly increased with additional gluing in all high-risk anastomoses [30]. Both systemic and local inhibition of matrix metalloproteinases, which play a central role in collagen remodeling, have been shown to improve bursting pressure in colonic anastomoses in rats [31,32,33]. In immunocompromised mice, insulin-like growth factor 1-coated sutures and systemic insulin-like growth factor-1 administration have been shown to improve anastomotic strength and hydroxyproline content in a mouse model of colitis [34,35].

In addition to intrinsic factors, extrinsic factors such as fibrin sealants are associated with hemostasis and wound healing [13]. Researchers exploring fibrin glue application have presented conflicting results. While Liu et al. did not detect AL in any of the 120 patients who underwent bariatric surgery with fibrin glue application, AL was detected in only one of 120 patients who did not receive fibrin glue by the same surgeon, revealing a non-significant difference [13]. It has been argued that pathogens such as *Pseudomonas aeruginosa*, *Serratia marcescens,* and *Enterococcus faecalis* activate the host-bacteria-mediated plasminogen system, causing collagenolysis and tissue invasion, leading to AL [36,37,38]. In a recent study, it was reported that tranexamic acid inhibited this process both in vitro and in vivo, and promising results showing AL prevention were described when this treatment was administered as an enema [39]. In an experimental study, a decrease in *Serratia marcescens* and *Pseudomonas aeruginosa* colonization and collagenase activity in anastomotic tissues was shown in mice receiving oral polyphosphate after colon surgery, indicating potential benefits [36]. It has also been demonstrated that suppression of collagenase production by *Enterococcus faecalis* prevented AL in an experimental model [37]. Huang et al. investigated a novel dopamine-conjugated xanthan gum adhesive (Dag-Xan) in a rat model. The authors reported that this polysaccharide derivative could facilitate angiogenesis and fibroblast infiltration, thus allowing healing of surgical anastomosis, which was also shown to result in improved burst pressure [12].

The healing process of intestinal anastomoses includes the classical “inflammation, proliferation, and remodeling” phases, but it differs from other tissues in some features including time and environmental interactions. At each stage of anastomosis healing, the initial inflammatory cells are replaced by collagen-producing fibroblasts, which strengthen the anastomosis through downstream activation, primarily via cytokines and growth factor release [28]. There is no doubt that inflammation plays a role in the occurrence of AL. However, the exact impact and the cellular and molecular aspects are unknown [27]. Cells that play a role in the early stages of inflammation are innate immune cells [40]. Neutrophils are glycolytic, able to function in low oxygen conditions. This may adversely affect the wound healing process due to the continuation of inflammatory activity under oxygen deprivation [27]. However, since inflammation and wound healing are intertwined processes, clear conclusions about whether suppressing inflammation provides a beneficial or harmful effect cannot be drawn. As a matter of fact, studies utilizing anti-inflammatory treatments have presented conflicting results, with suggestions that tissue response to various inflammatory factors could yield different outcomes [39,41,42,43,44]. We used an experimental model including both complete anastomosis and incomplete anastomosis. Our main findings showed that papaverine had no reasonable positive or negative effect on the third day, while it could have a suppressive role on inflammation on the seventh day at the site of anastomosis, especially in the complete anastomosis group. Based on these results, a definite conclusion about the effect of papaverine on AL cannot be arrived upon. Given the complexity of factors affecting the occurrence and severity of AL and the uncertain role of suppressing inflammation, more research is needed to ascertain the how papaverine can impact wound healing and anastomosis [8].

### Strengths and Limitations

To our knowledge, this study represents the first investigation of the impact of a single topically applied dose of papaverine on colon anastomosis healing. However, certain limitations need to be taken into account when interpreting the findings. As an experimental study, the direct applicability of the results to humans cannot be conclusively determined. Additionally, assessing the effects of a drug on normal anastomosis is challenging since rats typically do not exhibit considerable variations in anastomotic healing [1]. As an inevitable result of being an experimental study, the relatively small number of subjects may have limited statistical analyses. Only the properties of the anastomotic tissue in the first 7 days were investigated, and this period may be insufficient to gain insights on the whole picture due to the timewise variations in the timing of AL and the healing process. That is, while AL occurs most frequently in the first few days following surgery [9], wound healing takes longer [25]; therefore, the examination of factors associated with healing characteristics may not be representative of the exact nature of AL development.

## 5. Conclusions

To summarize, the findings of this study indicate that a single dose of topical papaverine administered to the anastomosis site did not significantly affect wound healing parameters on day 3 or day 7. However, it did show improvements in Ehrlich–Hunt score, inflammation score, and neutrophil score on day 7 in the complete anastomosis group, as well as in necrosis score on the seventh day in the incomplete anastomosis group. While topical papaverine demonstrated a tendency to reduce inflammation in the anastomotic wound area, further comprehensive studies are necessary to determine its impact on anastomotic wound healing and the occurrence of anastomotic leakage.

## Figures and Tables

**Table 1:** Outcomes of complete anastomosis on the third day with regard to papaverine use.

	Papaverine Use		*p*
	Yes (n = 8)	No (n = 8)	
Weight, g	319 (305–330)	336.5 (326–340)	0.002
Bursting pressure, mmHg	18.5 (4–34)	15 (9–21)	0.399
Hydroxyproline concentration, ng/mL	103.53 (81.18–172.76)	97.49 (52.72–141.19)	0.172
Ehrlich–Hunt score	9 (7–12)	9.5 (7–12)	0.787
Inflammation			
1	0 (0.0%)	0 (0.0%)	1.000
2	0 (0.0%)	0 (0.0%)	
3	2 (25.0%)	2 (25.0%)	
4	6 (75.0%)	6 (75.0%)	
Fibroblast			
0	2 (25.0%)	1 (12.5%)	1.000
1	4 (50.0%)	5 (62.5%)	
2	2 (25.0%)	2 (25.0%)	
3	0 (0.0%)	0 (0.0%)	
4	0 (0.0%)	0 (0.0%)	
Neoangiogenesis			
0	2 (25.0%)	1 (12.5%)	1.000
1	4 (50.0%)	5 (62.5%)	
2	2 (25.0%)	2 (25.0%)	
3	0 (0.0%)	0 (0.0%)	
4	0 (0.0%)	0 (0.0%)	
Necrosis			
0	0 (0.0%)	0 (0.0%)	1.000
1	0 (0.0%)	0 (0.0%)	
2	1 (12.5%)	0 (0.0%)	
3	5 (62.5%)	6 (75.0%)	
4	2 (25.0%)	2 (25.0%)	
Collagen deposition			
0	5 (62.5%)	6 (75.0%)	1.000
1	3 (37.5%)	2 (25.0%)	
2	0 (0.0%)	0 (0.0%)	
3	0 (0.0%)	0 (0.0%)	
4	0 (0.0%)	0 (0.0%)	
Neutrophil			
1	0 (0.0%)	0 (0.0%)	1.000
2	0 (0.0%)	0 (0.0%)	
3	2 (25.0%)	2 (25.0%)	
4	6 (75.0%)	6 (75.0%)	
Lymphocyte			
1	3 (37.5%)	0 (0.0%)	0.097
2	5 (62.5%)	6 (75.0%)	
3	0 (0.0%)	2 (25.0%)	
4	0 (0.0%)	0 (0.0%)	

Data are given as median (minimum–maximum) for continuous variables due to non-normality of distribution and as frequency (percentage) for categorical variables.

**Table 2:** Outcomes of incomplete anastomosis on the third day with regard to papaverine use.

	Papaverine Use		*p*
	Yes (n = 8)	No (n = 8)	
Weight, g	322 (310–338)	323.5 (316–334)	0.875
Bursting pressure, mmHg	3.5 (0–7)	2.5 (0–4)	0.455
Hydroxyproline concentration, ng/mL	128.57 (120.66–205.44)	111.07 (63.57–175.63)	0.248
Ehrlich–Hunt score	9 (6–10)	8 (6–10)	0.448
Inflammation			
1	0 (0.0%)	0 (0.0%)	1.000
2	1 (12.5%)	1 (12.5%)	
3	2 (25.0%)	3 (37.5%)	
4	5 (62.5%)	4 (50.0%)	
Fibroblast			
0	1 (12.5%)	1 (12.5%)	1.000
1	6 (75.0%)	7 (87.5%)	
2	1 (12.5%)	0 (0.0%)	
3	0 (0.0%)	0 (0.0%)	
4	0 (0.0%)	0 (0.0%)	
Neoangiogenesis			
0	0 (0.0%)	1 (12.5%)	1.000
1	7 (87.5%)	7 (87.5%)	
2	1 (12.5%)	0 (0.0%)	
3	0 (0.0%)	0 (0.0%)	
4	0 (0.0%)	0 (0.0%)	
Necrosis			
0	0 (0.0%)	0 (0.0%)	0.549
1	0 (0.0%)	1 (12.5%)	
2	3 (37.5%)	1 (12.5%)	
3	2 (25.0%)	4 (50.0%)	
4	3 (37.5%)	2 (25.0%)	
Collagen deposition			
0	7 (87.5%)	5 (62.5%)	0.569
1	1 (12.5%)	3 (37.5%)	
2	0 (0.0%)	0 (0.0%)	
3	0 (0.0%)	0 (0.0%)	
4	0 (0.0%)	0 (0.0%)	
Neutrophil			
1	1 (12.5%)	0 (0.0%)	0.765
2	1 (12.5%)	1 (12.5%)	
3	1 (12.5%)	3 (37.5%)	
4	5 (62.5%)	4 (50.0%)	
Lymphocyte			
1	2 (25.0%)	7 (87.5%)	0.018
2	4 (50.0%)	0 (0.0%)	
3	2 (25.0%)	1 (12.5%)	
4	0 (0.0%)	0 (0.0%)	

Data are given as median (minimum–maximum) for continuous variables due to non-normality of distribution and as frequency (percentage) for categorical variables.

**Table 3:** Outcomes of complete anastomosis on the seventh day with regard to papaverine use.

	Papaverine Use		*p*
	Yes (n = 8)	No (n = 8)	
Weight, g	316 (307–332)	326.5 (312–338)	0.074
Bursting pressure, mmHg	153.5 (141–214)	154.5 (110–204)	0.345
Hydroxyproline concentration, ng/mL	204.05 (160.82–282.97)	192.86 (121.34–266.70)	0.227
Ehrlich–Hunt score	14.5 (13–17)	17.5 (16–19)	0.012
Inflammation			
1	0 (0.0%)	0 (0.0%)	0.026
2	1 (12.5%)	0 (0.0%)	
3	4 (50.0%)	0 (0.0%)	
4	3 (37.5%)	8 (100.0%)	
Fibroblast			
0	0 (0.0%)	0 (0.0%)	0.282
1	0 (0.0%)	0 (0.0%)	
2	0 (0.0%)	0 (0.0%)	
3	4 (50.0%)	1 (12.5%)	
4	4 (50.0%)	7 (87.5%)	
Neoangiogenesis			
0	0 (0.0%)	0 (0.0%)	0.282
1	0 (0.0%)	0 (0.0%)	
2	0 (0.0%)	0 (0.0%)	
3	4 (50.0%)	1 (12.5%)	
4	4 (50.0%)	7 (87.5%)	
Necrosis			
0	1 (12.5%)	0 (0.0%)	0.230
1	4 (50.0%)	2 (25.0%)	
2	3 (37.5%)	2 (25.0%)	
3	0 (0.0%)	3 (37.5%)	
4	0 (0.0%)	1 (12.5%)	
Collagen deposition			
0	0 (0.0%)	0 (0.0%)	1.000
1	0 (0.0%)	0 (0.0%)	
2	0 (0.0%)	0 (0.0%)	
3	6 (75.0%)	7 (87.5%)	
4	2 (25.0%)	1 (12.5%)	
Neutrophil			
1	1 (12.5%)	0 (0.0%)	0.041
2	2 (25.0%)	1 (12.5%)	
3	3 (37.5%)	0 (0.0%)	
4	2 (25.0%)	7 (87.5%)	
Lymphocyte			
1	0 (0.0%)	0 (0.0%)	0.674
2	4 (50.0%)	3 (37.5%)	
3	2 (25.0%)	4 (50.0%)	
4	2 (25.0%)	1 (12.5%)	

Data are given as median (minimum–maximum) for continuous variables due to non-normality of distribution and as frequency (percentage) for categorical variables.

**Table 4:** Outcomes of incomplete anastomosis on the seventh day with regard to papaverine use.

	Papaverine Use		*p*
	Yes (n = 8)	No (n = 8)	
Weight, g	317 (303–338)	324.5 (302–337)	0.793
Bursting pressure, mmHg	120.5 (114–168)	116 (94–146)	0.207
Hydroxyproline concentration, ng/mL	234.12 (166.22–296.96)	231.38 (118.62–298.66)	1.000
Ehrlich–Hunt score	17 (11–18)	16 (10–19)	0.913
Inflammation, grade			
1	0 (0.0%)	0 (0.0%)	0.569
2	0 (0.0%)	1 (12.5%)	
3	1 (12.5%)	2 (25.0%)	
4	7 (87.5%)	5 (62.5%)	
Fibroblast, grade			
0	0 (0.0%)	0 (0.0%)	1.000
1	0 (0.0%)	0 (0.0%)	
2	1 (12.5%)	1 (12.5%)	
3	1 (12.5%)	2 (25.0%)	
4	6 (75.0%)	5 (62.5%)	
Neoangiogenesis, grade			
0	0 (0.0%)	0 (0.0%)	0.569
1	0 (0.0%)	0 (0.0%)	
2	1 (12.5%)	0 (0.0%)	
3	1 (12.5%)	3 (37.5%)	
4	6 (75.0%)	5 (62.5%)	
Necrosis, grade			
0	2 (25.0%)	0 (0.0%)	0.014
1	0 (0.0%)	3 (37.5%)	
2	6 (75.0%)	2 (25.0%)	
3	0 (0.0%)	3 (37.5%)	
4	0 (0.0%)	0 (0.0%)	
Collagen deposition, grade			
0	0 (0.0%)	0 (0.0%)	1.000
1	1 (12.5%)	1 (12.5%)	
2	0 (0.0%)	0 (0.0%)	
3	1 (12.5%)	2 (25.0%)	
4	6 (75.0%)	5 (62.5%)	
Neutrophil, grade			
1	0 (0.0%)	0 (0.0%)	0.713
2	0 (0.0%)	2 (25.0%)	
3	1 (12.5%)	1 (12.5%)	
4	7 (87.5%)	5 (62.5%)	
Lymphocyte, grade			
1	1 (12.5%)	0 (0.0%)	1.000
2	6 (75.0%)	7 (87.5%)	
3	1 (12.5%)	1 (12.5%)	
4	0 (0.0%)	0 (0.0%)	

Data are given as median (minimum–maximum) for continuous variables due to non-normality of distribution and as frequency (percentage) for categorical variables.

## Data Availability

Data are available on reasonable request to the corresponding author.
